# JZL184, A Monoacylglycerol Lipase Inhibitor, Induces Bone Loss in a Multiple Myeloma Model of Immunocompetent Mice

**DOI:** 10.1007/s00223-020-00689-0

**Published:** 2020-04-13

**Authors:** Silvia Marino, Giovana Carrasco, Boya Li, Karan M. Shah, Darren L. Lath, Antonia Sophocleous, Michelle A. Lawson, Aymen I. Idris

**Affiliations:** 1 Department of Oncology and Metabolism, Medical School, University of Sheffield, Beech Hill Road, Sheffield, S10 2RX UK; 2 IU School of Medicine, Division of Hematology/Oncology, Indiana University, Indianapolis, USA; 3 Department of Life Sciences, School of Sciences, European University Cyprus, 6 Diogenes Street, Nicosia, 1516 Cyprus

**Keywords:** MAGL, JZL184, Cannabinoid, Multiple myeloma, Bone, Cancer, Osteolysis, Osteoclast, Osteoblast

## Abstract

Multiple myeloma (MM) patients develop osteolysis characterised by excessive osteoclastic bone destruction and lack of osteoblast bone formation. Pharmacological manipulation of monoacylglycerol lipase (MAGL), an enzyme responsible for the degradation of the endocannabinoid 2-arachidonoyl glycerol (2-AG), reduced skeletal tumour burden and osteolysis associated with osteosarcoma and advanced breast and prostate cancers. MM and hematopoietic, immune and bone marrow cells express high levels of type 2 cannabinoid receptor and osteoblasts secrete 2-AG. However, the effects of MAGL manipulation on MM have not been investigated. Here, we report that treatment of pre-osteoclasts with non-cytotoxic concentrations of JZL184, a verified MAGL inhibitor, enhanced MM- and RANKL-induced osteoclast formation and size in vitro. Exposure of osteoblasts to JZL184 in the presence of MM cell-derived factors reduced osteoblast growth but had no effect on the ability of these cells to mature or form bone nodules. In vivo, administration of JZL184 induced a modest, yet significant, bone loss at both trabecular and cortical compartments of long bones of immunocompetent mice inoculated with the syngeneic 5TGM1-GFP MM cells. Notably, JZL184 failed to inhibit the in vitro growth of a panel of mouse and human MM cell lines, or reduce tumour burden in mice. Thus, MAGL inhibitors such as JZL184 can exacerbate MM-induced bone loss.

**Electronic supplementary material:**

The online version of this article (10.1007/s00223-020-00689-0) contains supplementary material, which is available to authorised users.

## Introduction

Bone disease is a serious complication in haematological malignancies such as multiple myeloma (MM). Approximately, 85% of MM patients develop bone disease characterised by excessive bone destruction, pain, fractures and lack of bone formation [1, 13, 29]. Thus, treatment strategies that aimed at reducing skeletal tumour burden, osteoclastic bone damage and bone pain would prove to be beneficial in terms of clinical outcomes in MM patients. Over recent years, there has been increasing interest in the therapeutic targeting of the endogenous cannabinoid (endocannabinoid) system for the management of skeletal-related events and a number of pre-clinical studies have implicated cannabinoid ligands and their receptors in regulation of bone cell activity, bone remodelling and bone pain [2, 19, 22, 25, 27, 28].

MM cells express high levels of the type 2 cannabinoid receptor (CB2), and immune cells are known to secrete the endocannabinoid 2-arachidonoyl glycerol (2-AG) [2, 9, 14, 21, 22, 43]. Thus far, limited research has been conducted on the therapeutic value of targeting the endocannabinoid system in the treatment of MM. In vitro studies have shown that exposure to plant-derived cannabidiol (CBD) and Δ9-tetrahydrocannabinol (THC), synthetic cannabinoid receptor agonist WIN-55,212–2 and to CB2 selective inverse agonists SR144528 and AM630 reduced the growth of various mouse and human MM cell lines [4, 14, 31, 36]. In vivo, Barbado and colleagues have observed that administration of WIN-55,212–2 reduced the growth of human U226 MM cells in immunodeficient mice [4]. Whilst these findings suggest pharmacological manipulation of cannabinoid receptors reduces MM cell growth, little is known about whether targeting the endocannabinoid system could be of therapeutic value in the reduction of skeletal complications associated with MM.

Monoacylglycerol lipase (MAGL) is an enzyme that is responsible for the degradation of the endocannabinoid 2-AG [15, 32, 40]. Previous studies have shown that 2-AG is secreted by bone cells at levels similar to those detected in the brain [2, 41–45]. Recent work carried out in our laboratories has demonstrated that administration of JZL184, a verified MAGL inhibitor, in mice protected against osteolytic bone damage induced by solid tumours of prostate, breast and bone origin, namely osteosarcoma [27]. Detailed examination of the effects of JZL184 in healthy and cancer bearing mice have led us to attribute the osteoprotective effects of this agent to its ability to reduce the growth of solid tumours in the skeleton rather than inhibition of osteoclastic bone resorption or stimulation of bone formation [27]. Intrigued by the high levels of expression of CB2 receptors by MM, we hypothesised that JZL184 could be of value in reducing MM-induced bone cell activity and osteolysis. Using a pharmacological approach that utilises data from experiments in cultures of osteoclasts, osteoblasts and MM cells and immunocompetent mice, we provide evidence that JZL184 enhanced MM-induced osteoclastogenesis in vitro and induced osteolytic bone loss in immunocompetent mice inoculated with the syngeneic murine 5TGM1-GFP cells without affecting tumour burden.

## Materials and Methods

### Reagents and Cells

The MAGL inhibitor JZL184 was purchased from Tocris Biosciences (Bristol, UK). Human JJN3 (DSMZ, Germany) and U266 (LGC Standards, UK), murine 5TGM1 (gift from Dr Oyajobi, University of Texas, San Antonio, USA), mouse RAW 264.7 macrophage and human osteoblast-like cells Saos-2 were originally purchased from ATCC (Manassas, VA). The minimum essential mediums (MEM) alpha(a)-MEM, delta(D)-MEM and RPMI were obtained from Sigma-Aldrich (Dorset, UK). Receptor activator of NFκB ligand (RANKL) was a gift from Patrick Mollat (Galapagos SASU, France) [17].

### Osteoclast Culture

Mouse RAW 264.7 macrophage (pre-osteoclasts) were seeded into 96-well plates at 2 × 10^3^ cells per well in standard D-MEM for up to 6 days in the presence and absence of RANKL (100 ng/ml) and/or conditioned medium from 5TGM1-GFP MM cells (10% v/v). Mature osteoclasts and their precursors were identified by staining for Tartrate-Resistant Acid Phosphatase (TRAcP). Osteoclast area was visualised by phase contrast microscopy on Olympus ScanR microscope, and area was quantified by Image Analysis using ImageJ.

### TRAcP Staining

TRAcP staining was used to identify multi-nucleated osteoclasts [20]. Osteoclast cultures were fixed in 4% paraformaldehyde and incubated with naphthol-AS-BI-phosphate, pararosaniline and tartrate in acetate buffer (30 µM) at 37 °C for 45 min as previously described [20]. TRAcP positive cells with 3 or more nuclei were considered to be osteoclasts and manually counted on a Zeiss Axiovert light microscope using a 10 × objective lens.

### Osteoblast Culture

The human osteoblast-like cells Saos-2 were seeded into 12-well plates at 10 × 10^5^ cells per well in standard D-MEM supplemented with β-glycerol phosphate (10 μM) and L-ascorbic acid (50 µg/ml) for up to 10 days in the presence and absence of 5TGM1-GFP conditioned media (20% *v/v*). Osteoblast number, differentiation and bone nodule formation were assessed by AlamarBlue assay, alkaline phosphatase assay and alizarin red (ALZ) staining [39].

### Alkaline Phosphatase Assay

Alkaline phosphatase activity was used to assess osteoblast differentiation [39]. The human osteoblast-like cells Saos-2 were homogenised in alkaline phosphatase lysis buffer containing diethanolamine (1 M), magnesium chloride (MgCl_2_, 1 mM) Triton X100 (0.05% *v/v*) and cell lysate was mixed with an equal volume of 4-nitrophenyl phosphate disodium salt hexahydrate (4 mM). Absorbance was measured at 405 nm using a SpectraMax M5® microplate reader (Molecular Devices, USA).

### Alizarin Red Staining

Alizarin red (ALZ) staining was used to visualise and quantify bone nodule formation in osteoblast cultures [39]. The human osteoblast-like cells Saos-2 were fixed and immersed in alizarin red solution (40 mM, pH 4.2) for 20 min at room temperature on an orbital rotator. A destaining solution (10%, w/v) of cetylpyridinium chloride in sodium phosphate (10 mM) was added for 15 min, and absorbance (562 nm) was then measured using a Synergy HT plate reader (Bio Tek, USA).

### MM Cell Culture

Mouse 5TGM1-GFP, and human U266 and JJN3 MM cells were cultured in standard RPMI supplemented with 10% FCS, penicillin and streptomycin. For viability assay, mouse 5TGM1-GFP and human U266 and JJN3 MM cells were seeded into 96-well plates at 10 × 10^3^ cells per well in standard D-MEM supplemented with 10% FCS, penicillin and streptomycin for 24 h. Cultures were then treated with vehicle or test compounds for the desired period. For studies involving conditioned medium, 5TGM1-GFP cells were allowed to grow to 80% confluency and the medium was refreshed with serum-free RPMI. After 16 h, the conditioned medium was removed and filtered (0.22 μm filter diameter). Freshly prepared conditioned medium (20% v/v) was added to osteoclast and osteoblast cultures and their precursors in standard alpha-MEM supplemented [26].

### AlamarBlue Assay

AlamarBlue assay was used to measure cell viability [33]. Cultures were treated with vehicle (0.1% *v/v* DMSO/PBS) or JZL184 (0–100 μM) for the desired period and then incubated in AlamarBlue reagent (10% *v/v*, Thermofisher, UK) for 2 h. Fluorescence was measured at excitation of 530 nm and emission of 590 nm using a SpectraMax® M5 microplate reader (Molecular Devices, USA).

### Real-Time RT-PCR

Real-time RT-PCR (qPCR) is used to assess the expression of cannabinoid receptors of MAGL in bone and MM cells. Briefly, total mRNA was extracted using RNeasy (QIAGEN, Germantown, MD) per the manufacturer’s protocol and reverse-transcribed using High-capacity cDNA reverse transcription kit (Applied Biosystem, Foster City, CA) on a T100 Thermal Cycler (Bio-Rad Laboratories, Hercules, CA). Quantitative PCR was performed on a CFX96 Real-Time System (Bio-Rad Laboratories, Hercules, CA) using a SsoAdvanced SYBR Green Supermix (Bio-Rad Laboratories, Hercules, CA) and cDNA equivalent to 40 ng RNA in a 10 µl reaction according to the manufacturer’s instructions. For amplification of mouse MAGL (forward primer: 5′-CAGAGAGGCCAACCTACTTTTC-3′, reverse primer 5′-ATGCGCCCCAAGGTCATATTT-3′); mouse CB1 (forward primer: 5′-GACGGTGTTTGCCTTCTGTAG-3′, reverse primer 5′-GAGCATAGATGATGGGGTTCA-3′) and mouse CB2 (forward primer: 5′-GGCAGTGTGACCATGACCTT-3′, reverse primer 5′-GGTCAACAGCGGTTAGCAG-3′) were used. Relative expression was calculated using the comparative 2-ΔΔCt method, with actin used as a housekeeping gene.

### Animal Experiments

All procedures involving animals were approved by the UK Home Office and the University of Sheffield’s Animal Ethics Committee. Female C57BL/6KalWRij mice (8-week-old) received tail-vein injection of mouse 5TGM1-GFP MM cells (10^6^ cells/mouse) as previously described [36, 37]. On day 2, animals were divided into two groups (8 mice per group) and received intraperitoneal injection of either vehicle (Dimethyl sulfoxide (DMSO)/water, 1:10) or JZL184 (16 mg/kg, thrice-weekly) for 21 days. Treatment regime used was chosen based on previous in vivo studies, which demonstrated that this agent exerted anti-tumour and anti-metastatic effects in mice [18, 27]. Animals were euthanized 21 days post injection of 5TGM1-GFP cells and bone architecture were assessed by micro-computed tomography (microCT) [5]. Tumour burden was assessed by flow cytometry measuring the number of green fluorescent protein (GFP) positive 5TGM1 MM cells in bone marrow, and by weighing mouse spleen as previously described [23, 36–38].

### Micro-Computed Tomography

Cortical and trabecular bone parameters were measured at the left femur (400 slices distal of the growth plate) and proximal tibia (200 slices distal of the growth plate) using Skyscan 1172 microCT scanner (Bruker microCT, Belgium) set at 60 kV and 150 µA [5]. Regions of interest were reconstructed by NRecon software and analysed using CTAn software (Bruker microCT, Belgium) [5].

### Bone Histomorphometry

Bones were fixed and embedded in methyl-methacrylate as previously described [12]. Osteoclast and osteoblast parameters were quantified using TRAcP and toluidine blue staining, respectively [12].

### Statistical Analysis

Comparison between groups was assessed by Student’s *t* test or analysis of variance (ANOVA) followed by Dunnet’s post hoc test (GraphPad Prism for Apple Macintosh, version 8). A p-value of 0.05 or below was considered statistically significant.

## Results

### JZL184 Exerts a Biphasic Effect on RANKL and MM-Induced Osteoclast Formation in Vitro

**Fig. 1:**
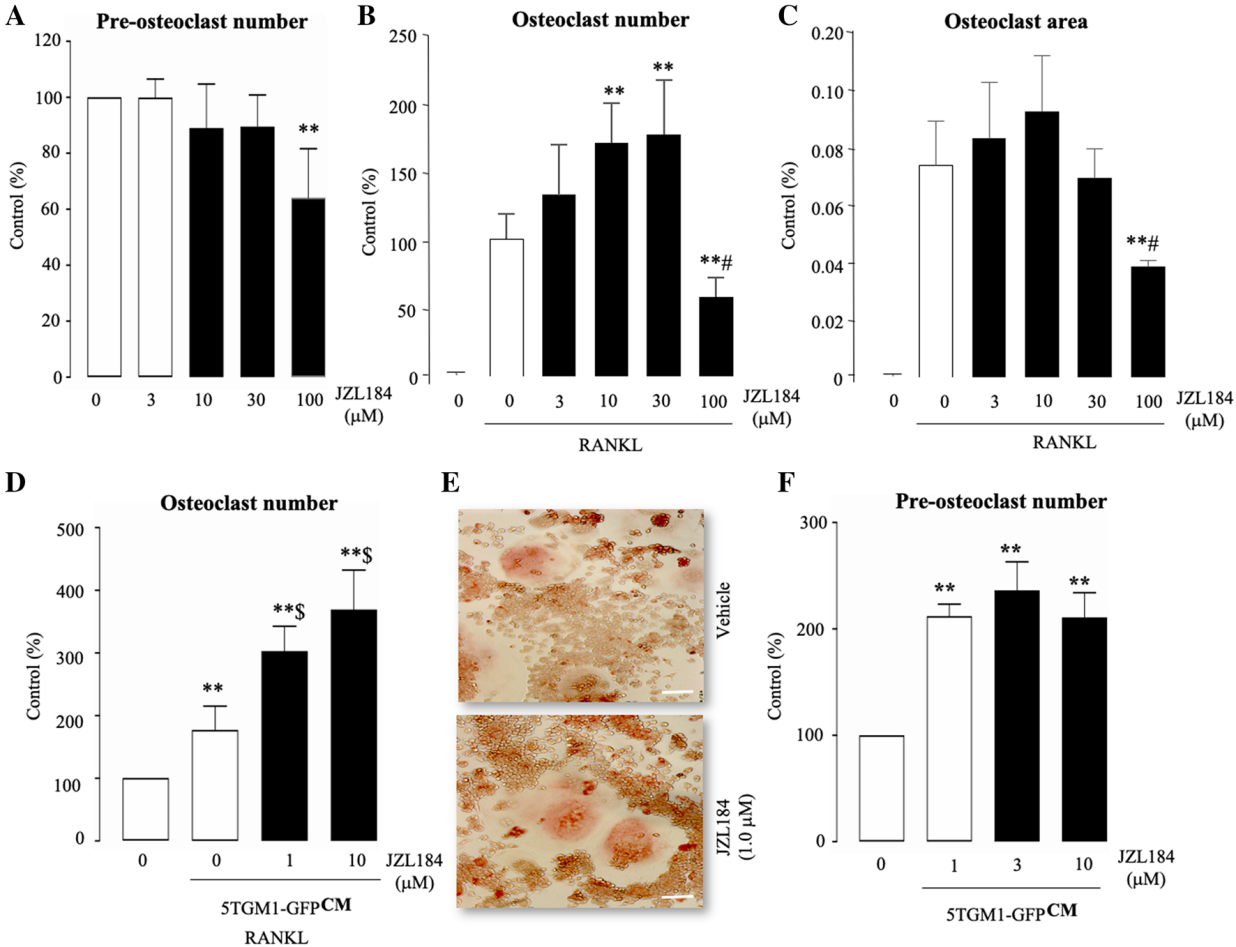
JZL184 enhances RANKL and MM-induced osteoclast formation. **a** In vitro cell viability and survival of mouse RAW 264.7 macrophage (pre-osteoclasts) treated with vehicle or JZL184 (0–100 μM) for 6 days, as assessed by AlamarBlue assay. **b** In vitro number of multi-nucleated osteoclasts with 3 or more nuclei in cultures of RANKL (100 ng/ml)-stimulated mouse RAW 264.7 mature osteoclasts treated with vehicle or JZL184 (0–100 μM) for 6 days as visualised by TRAcP staining. **c** Area of TRAcP positive osteoclasts in cultures of RANKL (100 ng/ml)-stimulated osteoclasts in mouse RAW 264.7 cultures treated with vehicle or JZL184 (0 –100 μM) for 6 days as measured by ImageJ. **d** In vitro multi-nucleated osteoclast number in cultures of RANKL-stimulated mouse RAW 264.7 pre-osteoclasts treated with vehicle or JZL184 (0–10 μM) for 7 days in the presence and absence of conditioned medium from 5TGM1-GFP MM cells (CM, 10% *v/v*), as visualised by TRAcP staining. **e** Representative photomicrographs of multi-nucleated osteoclasts from the experiment described in panel d. **f** In vitro viability and survival of osteoclast precursors in the experiment described in panels d and e, as assessed by AlamarBlue assay. Values are mean ± SD: **p < 0.01 from vehicle; # *p* < 0.05 from other JZL184 treated cultures; $ *p* < 0.05 from vehicle plus 5TGM1-GFP conditioned medium

The interactions between tumour cells and the bone marrow environment play critical roles in the development and progression of cancer-associated bone disease including MM [1, 13, 29]. We have recently reported that JZL184 exerts a biphasic effect on osteoclastogenesis in the presence and absence of osteotropic prostate and breast cancer cells, as well as osteosarcoma cells [27]. In view of this, we first assessed the effect of JZL184 (0–100 µM) on the ability of mouse RAW 264.7 macrophage cells (pre-osteoclasts) to survive and to form mature osteoclasts in the presence of RANKL. As shown in Fig. 1, panel A, JZL184 had no effect on the viability of pre-osteoclasts at a concentration range of 3 to 30 µM. At this concentration range, JZL184 enhanced RANKL-induced osteoclast number in cultures of mouse RAW 264.7 pre-osteoclasts (Fig. 1b, *p* < 0.01). Of note, exposure to JZL184 at 100 µM inhibited the ability of RAW 264.7 pre-osteoclasts to survive and form multi-nucleated osteoclasts (Fig. 1b) (*p* < 0.01). We attempted to study the effect of JZL184 on osteoclast activity by measuring the area of osteoclasts with 10 or more nuclei. As shown in Fig. 1c, exposure to JZL184 only inhibited osteoclast area at concentration of 100 µM (*p* < 0.01). Next, we went on to study the effects of JZL184 on MM-induced osteoclastogenesis. As shown in Fig. 1, panels d, e, conditioned medium (20% *v/v*) from mouse 5TGM1-GFP MM cells markedly increased osteoclast number in RANKL-stimulated RAW 264.7 macrophages and this effect was significantly enhanced by JZL184 (1–10 µM) (*p* < 0.01). In addition, JZL184 had no effects on MM-induced growth of pre-osteoclast at these concentrations (Fig. 1f).

### JZL184 Reduced Osteoblast Growth in the Presence of MM-Derived Factors

**Fig. 2:**
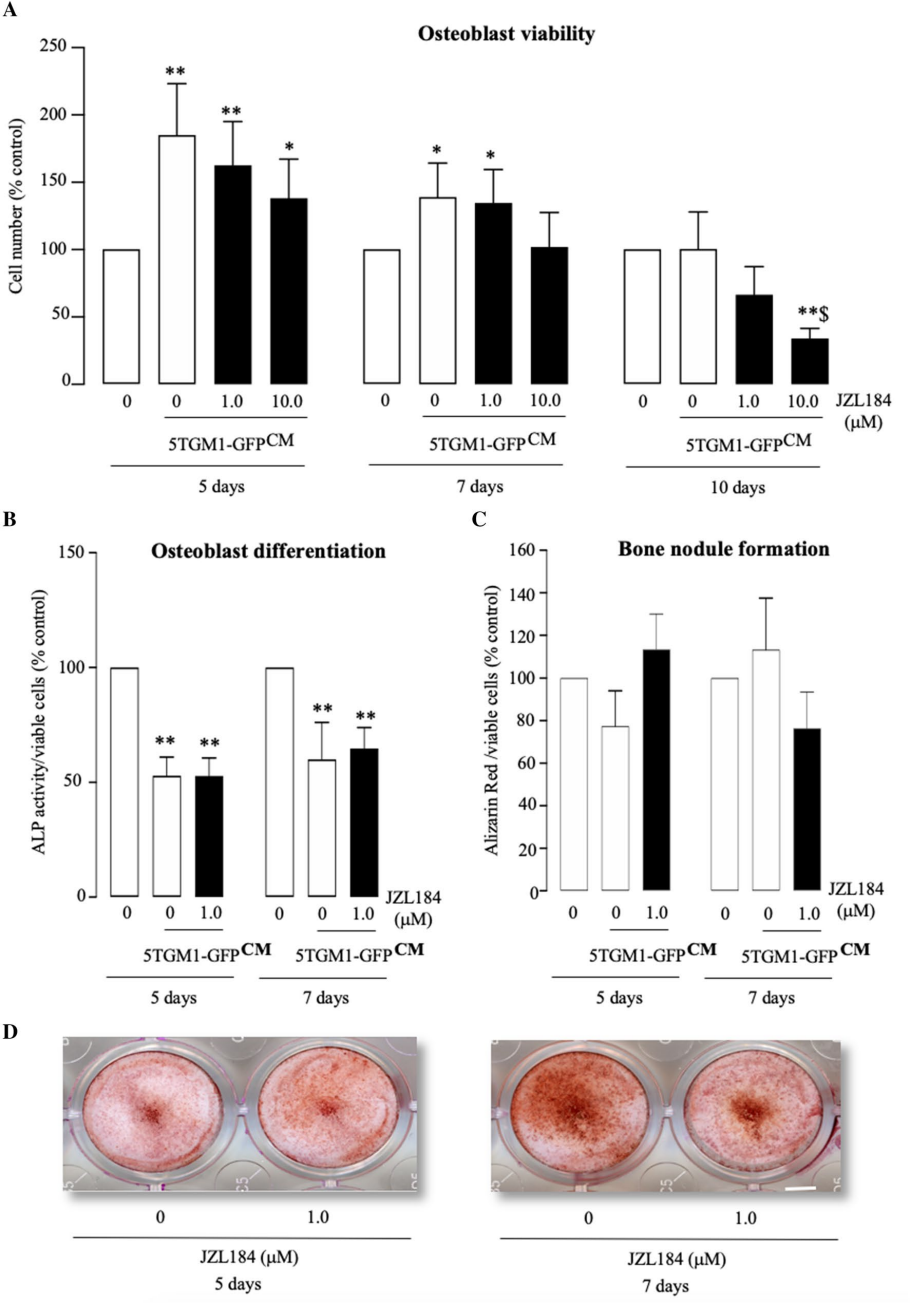
JZL184 reduced osteoblast growth in the presence of multiple myeloma-derived factors. **a** In vitro osteoblast viability in human osteoblast-like Saos-2 cultures exposed to standard or conditioned medium from 5TGM1-GFP MM cells (CM, 20% *v/v*) in the presence or absence of JZL184 (0–10 µM) for 5, 7 and 10 days, as assessed by AlamarBlue assay. **b**, **c** In vitro osteoblast differentiation (**b**) and bone nodule formation (**c**) in human osteoblast-like Saos-2 cultures exposed to standard or conditioned medium from 5TGM1-GFP MM cells (CM, 20% *v/v*) in the presence or absence of JZL184 (1 µM) for 5 and 7 days. Osteoblast viability (**a**), differentiation (**b**) and bone nodule formation (**c**) were assessed by AlamarBlue, Alkaline phosphatase and Alizarin Red assays, respectively. **d** Representative photomicrographs of bone nodule formation from the experiment described in panel c. Values are mean  ± SD. *p< 0.05 and **p < 0.01 from vehicle without conditioned medium; $ *p* < 0.05 from vehicle plus 5TGM1-GFP conditioned medium

JZL184 inhibited osteoblastic bone formation in mouse models of osteosarcoma [27]. Here, we first exposed the human osteoblast-like cells Saos-2 to a range of concentrations of JZL184 (0–100 µM), and we observed a significant inhibition of cell number within 3 days of exposure to a concentration 100 µM (Fig. S1). In view of this, we went on to test the effects of this agent on osteoblast proliferation, differentiation and bone nodule formation in the presence and absence of MM-derived factors at a concentration of 10 µM or lower. These experiments showed that conditioned medium (20% *v/v*) from mouse 5TGM1-GFP MM cells markedly increased the number of the human osteoblast-like cells Saos-2 after 5 and 7 days, and these effects were not significantly affected by exposure to JZL184 at concentrations up to 10 µM (Fig. 2a, left and middle panels). After 10 days, the effect of MM-derived factors on Saos-2 number was diminished, and JZL184 significantly reduced Saos-2 number at 10 µM (Fig. 2a, right panel) (*p* < 0.05). To study the effect of JZL184 on osteoblast maturation, we examined the effects of JZL184 (1 µM) on alkaline phosphatase activity (osteoblast differentiation marker) and bone nodule formation after 5 and 7 days of culture in the presence of MM conditioned medium. The treatment period and concentration were chosen on the basis that JZL184 exerted no detrimental effects on cell number or viability after 7 and 10 days of continuous exposure (Fig. 2a). As shown in Fig. 2b, conditioned medium (20% *v/v*) from mouse 5TGM1-GFP MM cells reduced alkaline phosphatase activity, indicative of reduced osteoblast differentiation, and this was not affected by JZL184 (1 µM) (*p* < 0.05). Interestingly, neither 5TGM1-GFP conditioned medium (20% *v/v*) nor JZL184 (1 µM) treatment affected the ability of the human osteoblast-like Saos-2 to form nodule after 5 and 7 days at the concentration tested (Fig. 2c). Representative photomicrographs of bone nodule formation from the experiments described are shown in Fig. 2, panel d.

### JZL184 Exerted a Biphasic Effect on MM Cell Number in Vitro

**Fig. 3:**
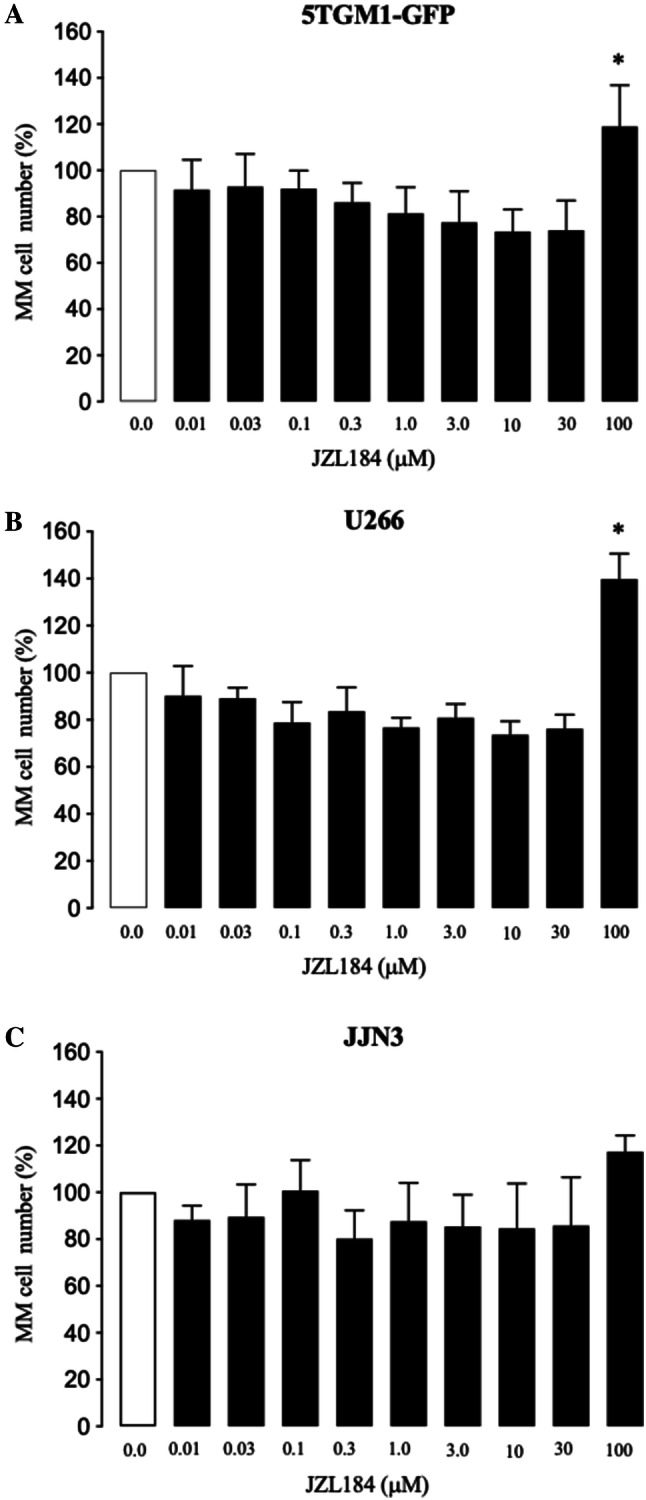
JZL184 exerted a biphasic effect on MM cell number in vitro. **a**–**c** In vitro viability of mouse 5TGM1-GFP (**a**) and human U266 (**b**) and JJN3 (**c**) MM cells exposed to vehicle (0.01% DMSO) or JZL184 (0–100 μM) for 48 h. Cell viability was assessed by AlamarBlue assay. Values are mean ± SD. *p < 0.05

Previous studies have shown that JZL184 reduced skeletal growth of osteosarcoma and osteotropic cancer cells of breast and prostate origin in vitro and in vivo [27]. In this study, we assessed the effects of JZL184 on the in vitro growth of a panel of mouse and human MM cell lines at concentrations up to 100 µM. As shown in Fig. 3, JZL184 (0–30 μM) exerted a modest inhibitory effect on the growth of mouse 5TGM1-GFP and human U266 and JJN3 MM cells (Fig. 3, panels a to c), a concentration range that caused a significant effect on osteoclastogenesis and osteoblast differentiation. At 100 µM, however, JZL184 significantly enhanced the growth of 5TGM1-GFP (Fig. 3a) and U266 (Fig. 3b) cells, and exerted a non-significant increase in the number of JJN3 cells (Fig. 3c).

### JZL184 Had No Effect Tumour Burden in Mice Bearing MM

**Fig. 4:**
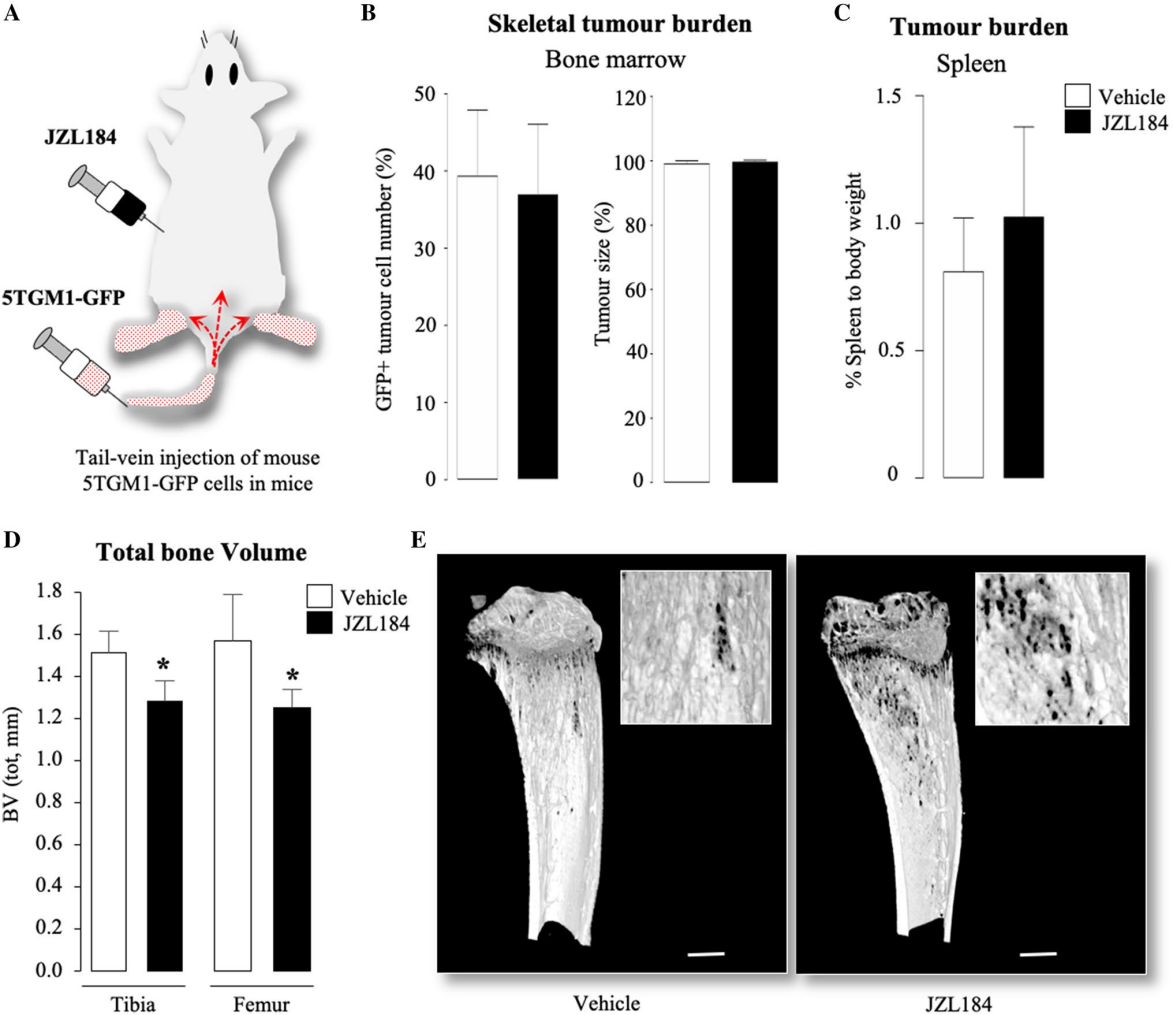
JZL184 induces bone loss without affecting tumour growth in mice bearing MM. **a** Graphic representation of tail-vein injection of mouse 5TGM1-GFP MM cells (10^6^ cells/mouse) into 8-week-old adult C57BL/6KalWRij mice treated with vehicle (0.01% DMSO) (*n* = 8 per group) or JZL184 (16 mg/kg, thrice-weekly) (*n* = 8) for 21 days. **b** Skeletal tumour burden as assessed by flow cytometry (left) and histological (right) analysis of GFP positive 5TGM1 MM cells in bone marrow (*n* = 8 per group), and presented as percentage of total cell count. **c** Tumour burden in spleen presented as percentage of spleen to body weight (*n* = 8 per group). **d** Total bone volume (BV, tot) at the tibia and femur of mice (*n* = 8 per group) from the experiment described in panels a–c, as assessed by microCT. **e** Representative photomicrographs of microCT scan of the tibial metaphysis of mice from the experiment described in panels a–d at low and high power (scale bar = 1 mm). Values are mean ± SD; *p < 0.05 versus vehicle

Next, we confirmed that the syngeneic MM cells 5TGM1-GFP express MAGL (Fig. S2, panel a), and went on to test the effects of JZL184 (16 mg/kg, thrice-weekly) on tumour burden in C57BL/6KalWRij female mice inoculated with 5TGM1-GFP MM cells (Fig. 4a). The main advantage of this model is it allowed us to examine the effects of JZL184 on the progression of MM in immunocompetent mice. The dosing regime of JZL184 have been chosen on the basis of previous mouse studies that have shown it reduced cancer-induced bone disease [27] and reduced tumour growth and metastasis [15, 34, 35]. As shown in Fig. 4b, administration of JZL184 had no effect on skeletal tumour burden as evident by the number of GFP positive MM cells in the bone marrow (Fig. 4b, left) and tumour size (Fig. 4b, right), when compared to vehicle (0.01% DMSO) treated mice. Similarly, JZL184 had no effect on tumour burden in the spleen when compared to vehicle-treated mice (Fig. 4c).

### JZL184 Reduced Trabecular and Cortical Bone Volume in Mice Bearing MM

**Fig. 5:**
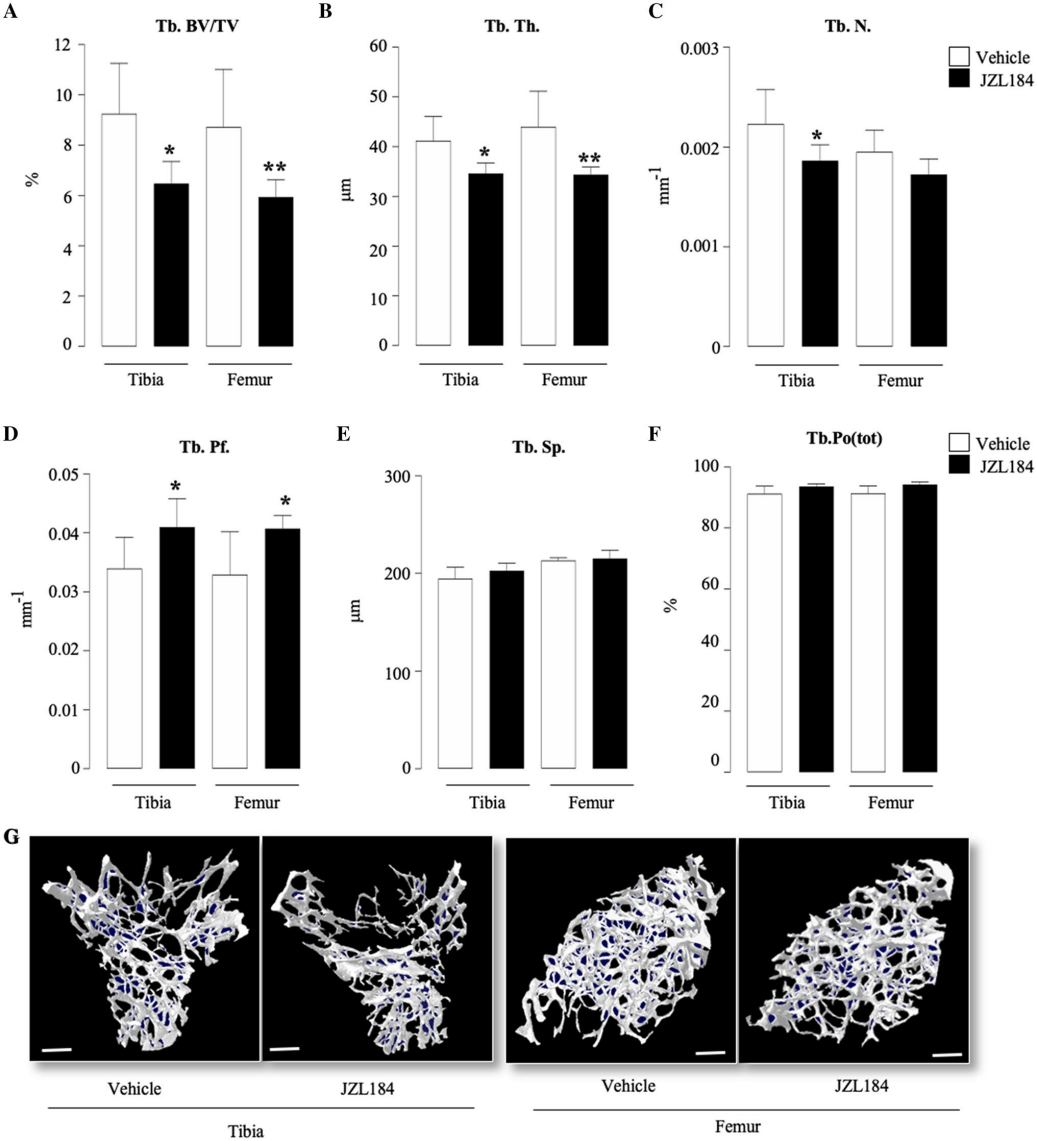
JZL184 induces trabecular bone loss in mice bearing MM. Trabecular bone parameters at the tibial and femoral metaphysis of 8 weeks old adult C57BL/6KalWRij mice treated with vehicle (0.01% DMSO) (*n* = 8) or JZL184 (16 mg/kg, thrice-weekly) (*n* = 8 per group) for 21 days post injection of mouse 5TGM1-GFP MM cells (10^6^ cells/mouse). Trabecular bone volume (BV/TV, panel** a**), trabecular thickness (Tb.Th, panel** b**), trabecular number (Tb.N, panel** c**), trabecular pattern factor (Tb. Pf, panel** d**), trabecular separation (Tb.S, panel** e**) and trabecular porosity (Ct.Po(tot), panel** f**) were assessed by microCT. **g** Representative microCT images of trabecular bone from tibiae and femur of mice described in panels a–f (scale bar = 1 mm). Values are mean ± SD; *p < 0.05 and **p < 0.01 versus vehicle

**Fig. 6:**
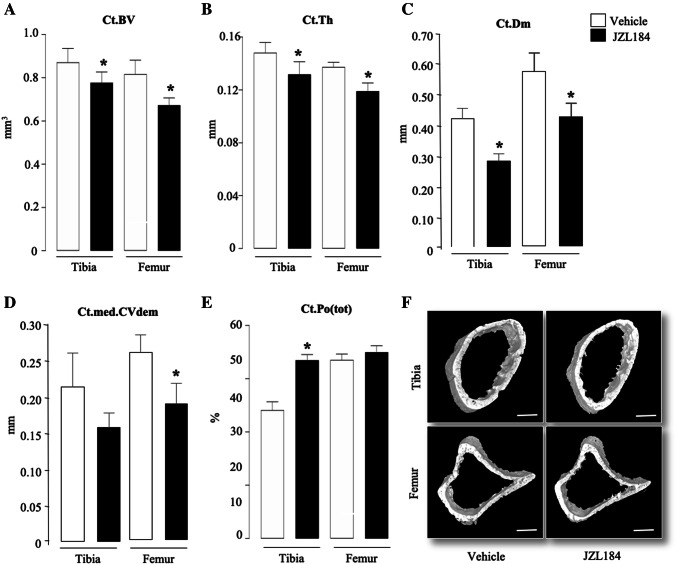
JZL184 induces cortical bone loss in mice bearing MM. Cortical bone parameters at the tibial and femoral metaphysis of 8-week-old adult C57BL/6KalWRij mice treated with vehicle (0.01% DMSO) (*n* = 8) or JZL184 (16 mg/kg, thrice-weekly) (*n* = 8) for 21 days post injection of mouse 5TGM1-GFP MM cells (10^6^ cells/mouse). Cortical bone volume (Ct.BV, panel** a**), cortical thickness (Ct.Th, panel** b**), cortical diameter (Ct.Dm, panel** c**), medullary cavity diameter (CtmedCVdem, panel** d**) and cortical porosity (Ct.Po(tot), panel** e**) were assessed by microCT. **f** Representative microCT images of cortical bone from tibiae and femur of the mice described in panels a–e (scale bar = 1 mm). Values are mean ± SD; *p < 0.05 versus vehicle

**Fig. 7:**
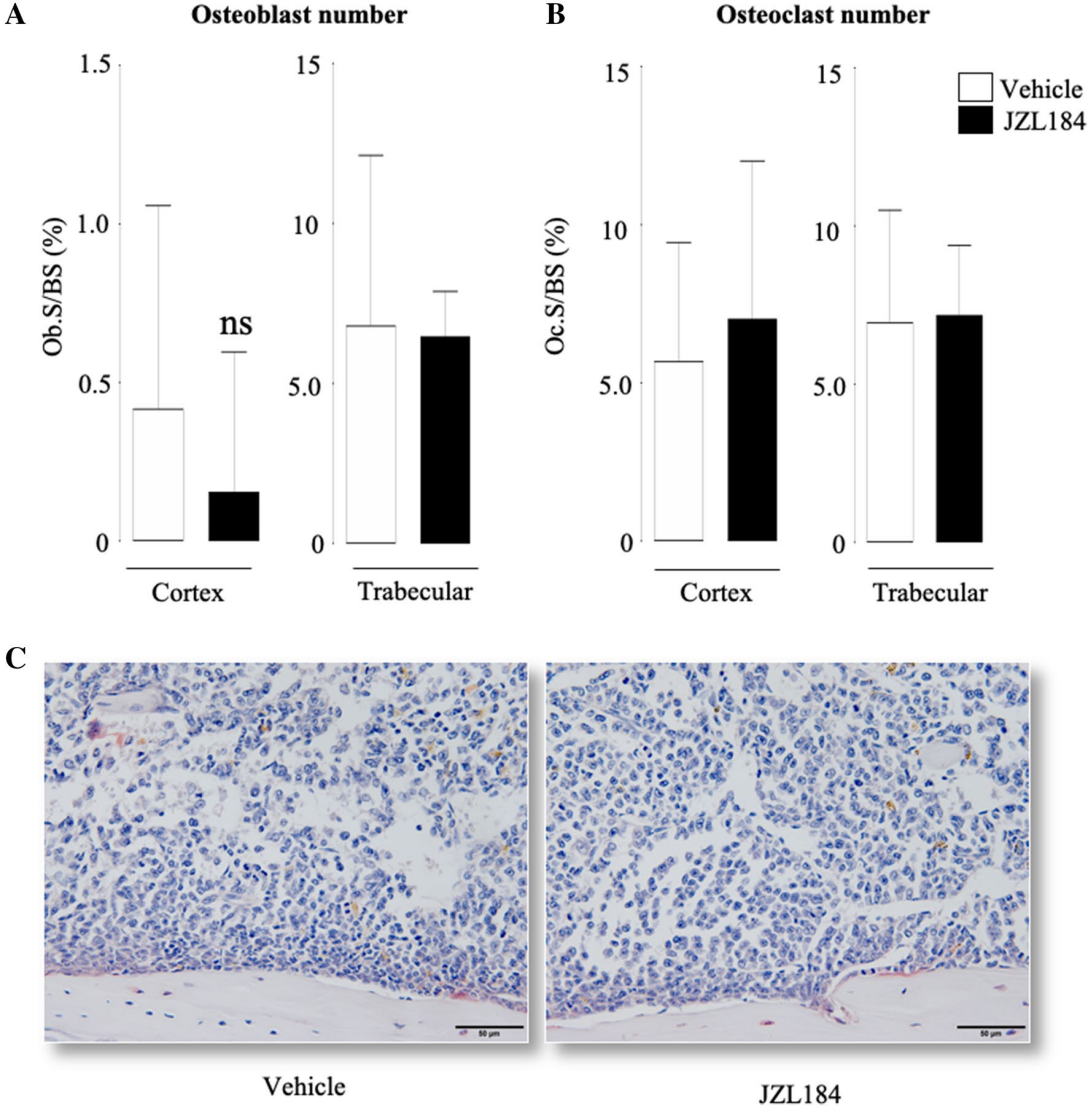
Effects of JZL184 on osteoblast and osteoclast number in mice bearing MM. In vivo percentage of osteoblasts (**a**, Ob.S/BS, %) and osteoclasts (**b**, Oc.S/BS, %) from the cortical and trabecular compartments of the tibial metaphysis of 8-week-old adult C57BL/6KalWRij mice treated with vehicle (0.01% DMSO) (*n* = 8) or JZL184 (16 mg/kg, thrice-weekly) (*n* = 8) for 21 days post injection of mouse 5TGM1-GFP MM cells (10^6^ cells/mouse). **c** Representative photomicrographs of histological sections from the experiment described. Scale bar = 50 µm. Values are mean ± SD; ns denotes not statistically significant

Detailed microCT analysis of the trabecular and cortical bone compartments of the long bones of the mice from the experiment described in Fig. 4a showed that administration of JZL184 (16 mg/kg, thrice-weekly) in immunocompetent mice bearing 5TGM1-GFP MM cells caused significant loss of total bone volume (Fig. 4d and e). Analysis of the trabecular compartment of these mice revealed significant reduction in bone volume (Tb.BV/TV, Fig. 5a) that is characterised by significant reduction in trabecular thickness (Tb.Th, Fig. 5b) and reduced trabecular connectivity as measured by the increase in trabecular pattern factor (Tb.Pf, Fig. 5d). Interestingly, we detected significant loss in trabecular number in the tibia—but not the femur—of these mice (*p* < 0.05, Tb.N, Fig. 5c). Additionally, we observed no changes in trabecular separation (Tb.Sp, Fig. 5e) or trabecular porosity (Tb.Po(tot), Fig. 5f). Representative microCT images of trabecular bone from tibiae and femurs of the mice described are shown in Fig. 5, panel g. In the cortical compartment, treatment with JZL184 reduced cortical bone volume (Ct.BV, Fig. 6a), cortical thickness (Ct.Th, Fig. 6b), cortical diameter (Ct.Dm, Fig. 6c) and medullary cavity diameter (CtmedCVdem, Fig. 6d) in both tibia and femur of mice bearing the mouse 5TGM1-GFP MM cells. Furthermore, JZL184 increased cortical porosity (Ct.Po(tot), Fig. 6e) at the tibia—but not at the femur—of these mice. Representative microCT images of cortical bone from tibiae and femurs of the mice described are shown in Fig. 6, panel f. Collectively, these results confirm that JZL184 induces bone loss in the MM mouse model described. Surprisingly, histological and histomorphometrical analysis of the tibial metaphysis revealed that JZL184 exerted no significant effects on the number of osteoclasts in both trabecular and cortical compartments (Fig. 7a). Histological and histomorphometrical analysis of the tibial metaphysis revealed a non-significant reduction in osteoblast number in the trabecular—but not cortical—compartment (Fig. 7a, *p* < 0.05). Analysis of osteoclast number in these sites showed that JZL184 exerted no significant effect (Fig.7b and c). Representative photomicrographs of histological sections from the experiment described are shown in Fig. 7, panel c.

### JZL184 Had No Effect on Body and Spleen Weight in Mice Bearing MM

**Fig. 8:**
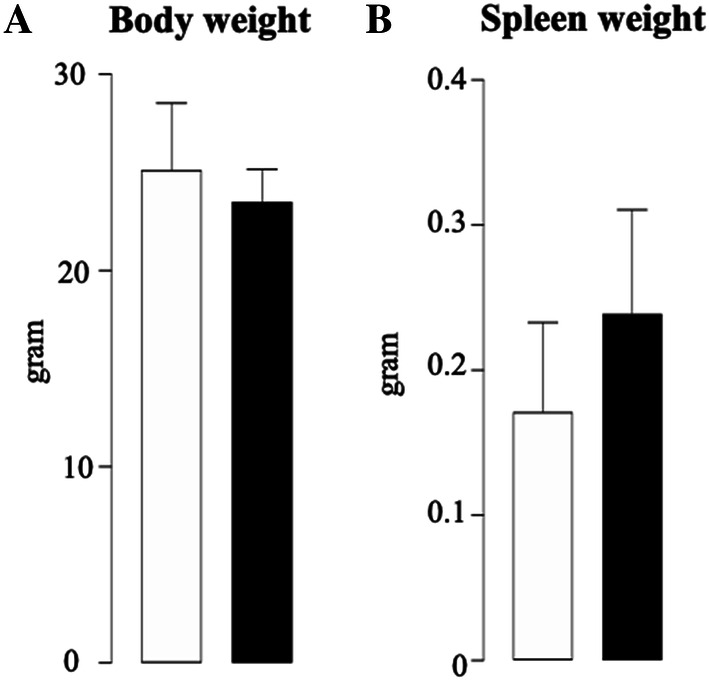
Effects of JZL184 on body or spleen weight in mice bearing MM. Total body (**a**) and spleen (**b**) weight in 8-week-old adult C57BL/6KalWRij mice treated with vehicle (0.01% DMSO) (*n* = 8) or JZL184 (16 mg/kg, thrice-weekly) (*n* = 8 per group) for 21 days post injection of mouse 5TGM1-GFP MM cells (10^6^ cells/mouse). Values are mean ± SD

Body weight is an important determinant of bone mass and previous studies have shown that manipulation of the endocannabinoid system affects appetite and body weight [7, 8, 10]. Here, we report that administration of JZL184 (16 mg/kg, thrice-weekly) had no effect on body (Fig. 8a) or spleen (Fig. 8b) weight in immunocompetent mice bearing the mouse 5TGM1-GFP MM cells when compared to vehicle-treated mice.

## Discussion

MM cells express the CB2 receptor, and immune and bone cells secrete the endocannabinoid 2-AG [2, 9, 14, 21, 22, 43]. Previous studies by Nomura et al*.* and our laboratories have shown that knockdown and pharmacological inhibition of MAGL—the enzyme that is responsible for the degradation of 2-AG [15, 32, 40]—inhibited the in vitro and in vivo growth of primary bone sarcoma and reduced the metastatic spread and bone damage associated with prostate and breast cancer [27, 32, 34]. In the present study, we report the effects of pharmacological manipulation of MAGL on the initiation and progression of tumour burden and bone disease associated with MM. Using a combination of in vitro studies in cultures of MM and bone cells and the murine 5TGM1-GFP model of MM, we observed that 5TGM1-GFP MM cells express MAGL but the exposure of these cells to the verified MAGL inhibitor JZL184 had no effect on their ability to grow in the bone marrow or spleen of immunocompetent mice. This finding contradicted previous published studies by our laboratories and others [27, 32, 34] that showed JZL184 reduced tumour growth in mouse models of osteosarcoma and prostate and breast cancers. Whist we cannot readily explain this, it is important to note that MM cells express MAGL and high levels of CB2 receptors [2, 9, 14, 21, 22, 43] and our present data showed that exposure to JZL184 at 100 µM significantly enhanced the in vitro growth of mouse 5TGM1 MM cells. Thus, we cannot exclude the possibility that MAGL and 2-AG expressed by these cells—as well as immune and/or bone cells—have no role in the tumour burden in the model described. Moreover, the anti-tumour effects of JZL184 that we and others have previously reported were observed in models of solid tumours [27, 32, 34]. Therefore, the fact that hematopoietic tumours including MM cells are anatomically different from solid tumours, could provide a plausible explanation for the lack of anti-tumour effect by JZL184 in our model. For example, hypoxia plays different roles in the progression of MM and solid tumours [16], and there is evidence to suggest that hypoxia-induced inhibition of the endocannabinoid system enhances glioblastoma cell growth [16]. Thus, it is likely that different hypoxic conditions in mice bearing MM and solid tumours might have affected the level tumour- and/or host-derived endocannabinoids by JZL184. Therefore, further in vivo studies are needed.

Osteolysis plays an important role in the pathogenesis of MM-associated bone disease [1, 13, 29]. In view of this, we examined the effects of JZL184 on osteolytic bone damage in mice bearing the syngeneic 5TGM1-GFP cells. This experiment revealed that administration of JZL184 induced a modest, yet significant, loss in total bone volume in the long bones of mice. Detailed microCT analysis showed that the bone-wasting effect of this agent was evident at both trabecular and cortical bone compartments of the tibia and femur of mice bearing 5TGM1-GFP MM cells. These findings are in broad agreement with our previous results that showed JZL184 induced bone loss in non-cancer bearing immunocompetent mice [27]. To gain an insight in the effects of JZL184 on bone cell activity in the presence and absence of MM cells, we first tested the effects of JZL184 on osteoclast formation and size in the presence of factors derived from 5TGM1-GFP MM cells. Our studies showed that JZL184 enhanced both osteoclast number and area induced by RANKL and conditioned medium from 5TGM1-GFP MM cells, indicative of stimulatory effects on osteoclast formation and size. It is important to note here that the cultures of osteoclasts and their precursor cells were exposed to JZL184 at concentrations that failed to affect cell viability or survival—thus excluding any cytotoxic effects. These results are consistent with our previous findings that showed that JZL184—and the endocannabinoid 2-AG—enhanced osteoclast formation at concentrations that failed to suppress M-CSF dependent bone marrow pre-osteoclasts [27]. Quantitative assessment of cannabinoid receptor expression showed that the mouse RAW 264.7 pre-osteoclasts used in the present studies express the cannabinoid receptor CB2 (Fig. S2, panel B), further implicating the endocannabinoid systems in these effects. In contrast to these findings and our present in vitro data in osteoclast cultures, JZL184 had no effect in osteoclast number in trabecular bones of mice bearing the syngeneic 5TGM1 MM cells. Of note, the mice in the present experiment suffered significant loss in trabecular bone and thus only small number of osteoclasts were present. In the cortical compartment, we observed no significant effect in osteoclast number but we noted a non-significant reduction in osteoblast number.

Osteoblasts produce the endocannabinoid 2-AG and we reported that JZL184 exerted a paradoxical effect on osteoblast differentiation and activity in the presence and absence of osteosarcoma and osteotropic prostate and breast cancer cells. At a concentration range that JZL184 enhanced osteoclast formation in vitro, it had no effect on the ability of the human osteoblast-like Saos-2 to grow, mature and form bone nodules in the presence of factors derived from 5TGM1-GFP MM cells.

Similarly, JZL184 failed to affect osteoblast number and activity in mice bearing 5TGM1-GFP MM cells. Interestingly, in vitro exposure to JZL184 at 100 µM for 3 days and 10 µM for 10 days reduced the number of the human osteoblast-like cells Saos-2. Whist it is reasonable to attribute this effect to cytotoxicity, Saos-2 cells secrete 2-AG in culture [27], thus it is possible that Saos-2-derived 2-AG—in cooperation with a cocktail of other mediators and signalling pathways [6, 11, 24, 30]—might have contributed to these effects. The support for this hypothesis comes from the observation that administration of JZL184 caused a non-significant reduction in osteoblast number in the trabecular—but not cortical—compartment of mice bearing MM cells.

Collectively, the results presented in this study extend our and others previous findings on the role of the endocannabinoid system in the skeleton [3, 19, 27, 28], and support the hypothesis that MAGL inhibitors such as JZL184 are ineffective in reducing neither tumour growth nor osteolytic bone damage caused by haematological malignancies such as MM. The ability of JZL184 to enhance osteoclast number and reduce osteoblast number in vitro provide a tentative mechanism for the significant loss in trabecular and cortical bone observed in mice bearing 5TGM1-GFP MM cells. When combined with previous studies that showed MAGL inhibitors such as JZL184 induce bone loss in healthy mice but reduce bone damage by inhibiting the growth of solid tumours [27], our current data reveal a complex role of MAGL and the body’s own cannabinoid system in the regulation of bone remodelling in solid [27, 32, 34] versus haematological ‘liquid’ malignancies such as MM.

## Electronic supplementary material

Below is the link to the electronic supplementary material.

Supplementary file1 (JPG 75 kb)—Fig. S1. Effects of JZL184 on osteoblast viability. In vitro osteoblast number in human osteoblast-like Saos-2 cultures treated with JZL184 (0 - 100μM) for 3 days, as assessed by AlamarBlue assay. Values are mean ± SD. * p < 0.05 versus vehicle.

Supplementary file2 (TIFF 1243 kb)—Fig. S2. Expression of MAGL and cannabinoids receptors. (A) mRNA expression of MAGL in mouse 5TGM1-GFP MM cells. (B) mRNA expression of CB1 and CB2 cannabinoid receptors in mouse RAW 264.7 macrophage (pre-osteoclasts). Relative (rel) expression was calculated using the comparative 2-ΔΔCt method, with actin used as a housekeeping gene. Values are mean ± SD.

## Abbreviations

MAGL: Monoacylglycerol lipase
MM: Multiple Myeloma
2-AG: 2-Arachidonoyl glycerol
NFκB: Nuclear factor kappa-B
RANK: Receptor activator of NFκB
RANKL: RANK ligand
M-CSF: Macrophage colony stimulating factor
DMSO: Dimethyl sulfoxide
microCT: Micro-computed tomography
TRAcP: Tartrate-resistant acid phosphatase
Alk Phos: Alkaline phosphatase
ALZ: Alizarin red
GFP: Green fluorescent protein
BM: Bone marrow
ANOVA: Analysis of variance
SD: Standard deviation
mm: Millimetre
MEM: Minimum essential medium
