# Supplementary figures and images for: JZL184, A Monoacylglycerol Lipase Inhibitor, Induces Bone Loss in a Multiple Myeloma Model of Immunocompetent Mice
Source: Calcif Tissue Int. 2020 Apr 13;107(1):72–85. doi: 10.1007/s00223-020-00689-0 (PMC7271071; doi:10.1007/s00223-020-00689-0)

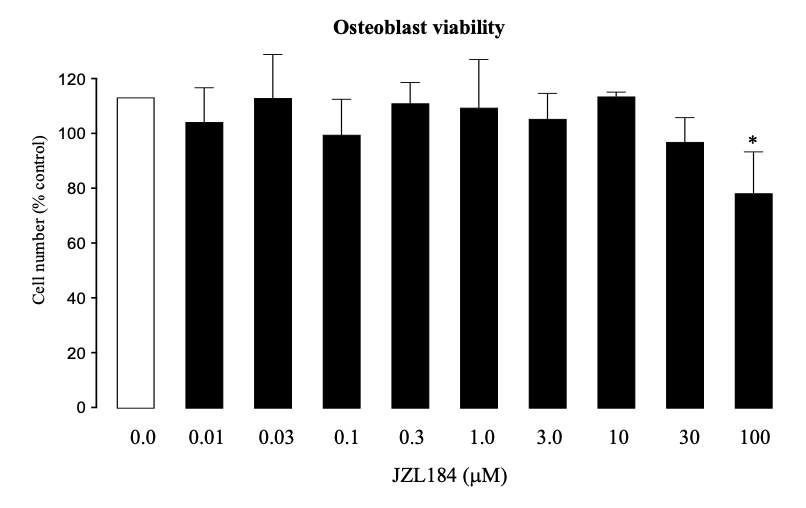

Supplement: Supplementary file 1 — Supplementary file1 (JPG 75 kb)—Fig. S1. Effects of JZL184 on osteoblast viability. In vitro osteoblast number in human osteoblast-like Saos-2 cultures treated with JZL184 (0 - 100μM) for 3 days, as assessed by AlamarBlue assay. Values are mean ± SD. * p < 0.05 versus vehicle. [file 223_2020_689_MOESM1_ESM.jpg]

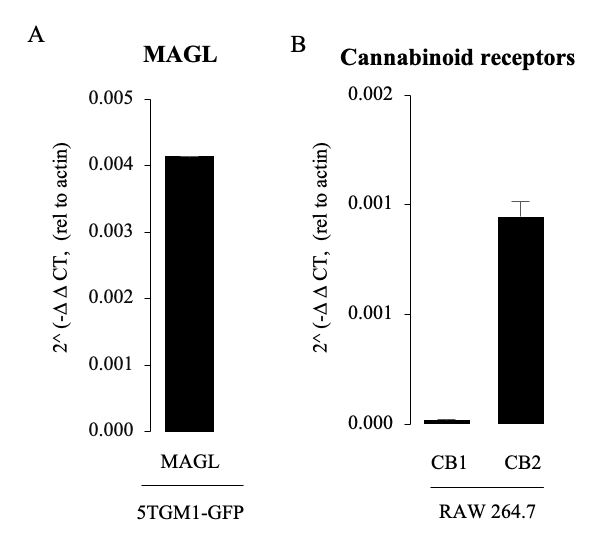

Supplement: Supplementary file 2 — Supplementary file2 (TIFF 1243 kb)—Fig. S2. Expression of MAGL and cannabinoids receptors. (A) mRNA expression of MAGL in mouse 5TGM1-GFP MM cells. (B) mRNA expression of CB1 and CB2 cannabinoid receptors in mouse RAW 264.7 macrophage (pre-osteoclasts). Relative (rel) expression was calculated using the comparative 2-ΔΔCt method, with actin used as a housekeeping gene. Values are mean ± SD. [file 223_2020_689_MOESM2_ESM.tiff]
